# Adenoid Cystic Carcinoma of the Uterine Cervix: A Report of 2 Cases

**DOI:** 10.1155/2017/8401741

**Published:** 2017-02-28

**Authors:** Meryem Rais, Jinane Kharmoum, Soumaya Ech-Charif, Basma El Khannoussi

**Affiliations:** Department of Pathology, National Institute of Oncology, Rabat, Morocco

## Abstract

Adenoid cystic carcinoma is malignant tumor that exceptionally occurs in the uterine cervix. It is mostly seen in postmenopausal women and has an aggressive clinical course. We report two cases of an adenoid cystic carcinoma associated with a high grade squamous intraepithelial lesion and invasive squamous cell carcinoma of the uterine cervix and discuss briefly its clinical and pathological characteristics.

## 1. Introduction

Adenoid cystic carcinoma (ACC) is a rare malignant tumor that has a distinctive histological appearance; it is most commonly seen in salivary glands but can involve many sites that have a secretory gland component [1]. The first case of ACC of the cervix was reported as a cylindroma in 1949 [2]. It is a very rare tumor, accounting for less than 1% of all cervical carcinomas [3]. It has mostly been reported in small case series and single case reports [4, 5]. ACC of the cervix is usually seen in postmenopausal women [6]. Very rare cases have been reported in women under the age of 40 years [7], and some authors suggested the association between ACC, high parity, and black race [8]. On histopathologic examination, this tumor resembles those located more commonly in the salivary glands or respiratory tract [6]. ACC is generally a locally aggressive tumor and has a high propensity for local recurrence and distant metastasis [9]. Thus, it needs to be differentiated from other less aggressive tumors. Immunohistochemically, ACC cells stain positively with broad spectrum cytokeratin, CAM 5.2, CK7, CD 117, and EMA. CEA may also be expressed. Nevertheless, cervical ACC contains few or no myoepithelial cells, which could distinguish this tumor from ACC of other sites. In fact, S-100 protein reactivity occurs in some cases while others are unreactive or just focally and weakly reactive [6, 10–12]. Also, ACC has rarely been reported to be associated with intraepithelial squamous neoplasia, invasive squamous cell carcinoma (SCC), adenocarcinoma, and sarcoma in the uterine cervix [11, 12].

## 2. Case Presentations

**Case 1 .**

A 74-year-old woman was admitted in our hospital with the complaint of spontaneous vaginal bleeding for 2 months before consultation. The patient had a past medical history of surgery for uterine leiomyoma. She had been on medication for hypertension and diabetes. She had no history of hormone replacement therapy.

Per speculum examination showed a raised lesion located on the posterior lip of the cervix. Bimanual rectovaginal examination showed no parametrial involvement. Pelvic ultrasound and Chest X-ray revealed no anomaly.

A cervical pap smear showed atypical squamous cells, suspicious of high grade squamous intraepithelial lesion.

**Figure 1:**
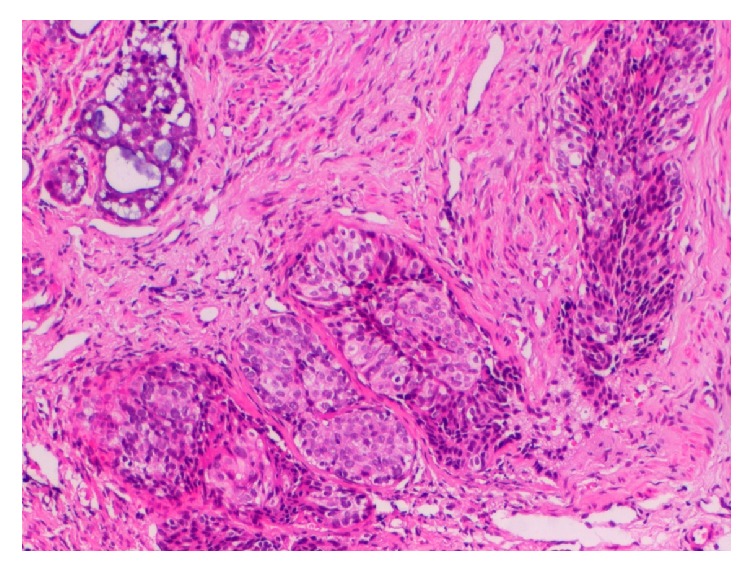
Photomicrograph of biopsy specimen of Case 1, showing the squamous cell carcinoma component (middle) and the adenoid cystic carcinoma component (upper left) (Hematoxylin and Eosin ×100).

**Figure 2:**
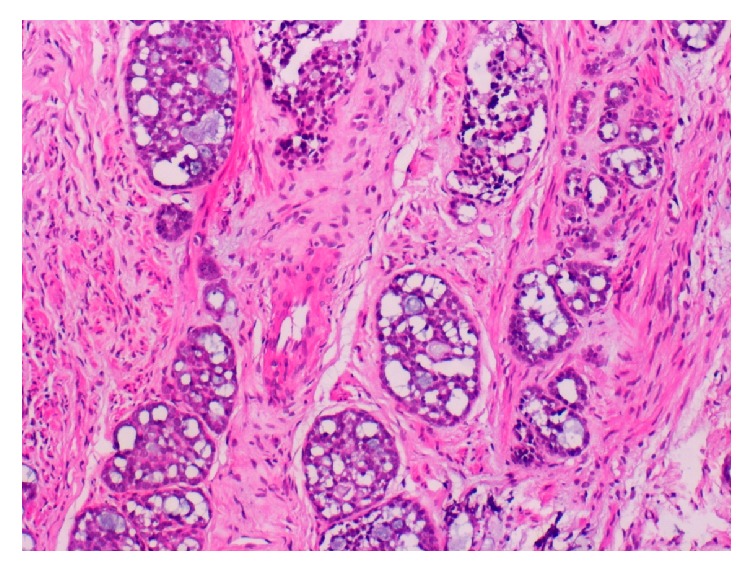
Photomicrograph of biopsy specimen of Case 1, showing the adenoid cystic carcinoma component, cribriform pattern (HE ×400).

**Figure 3:**
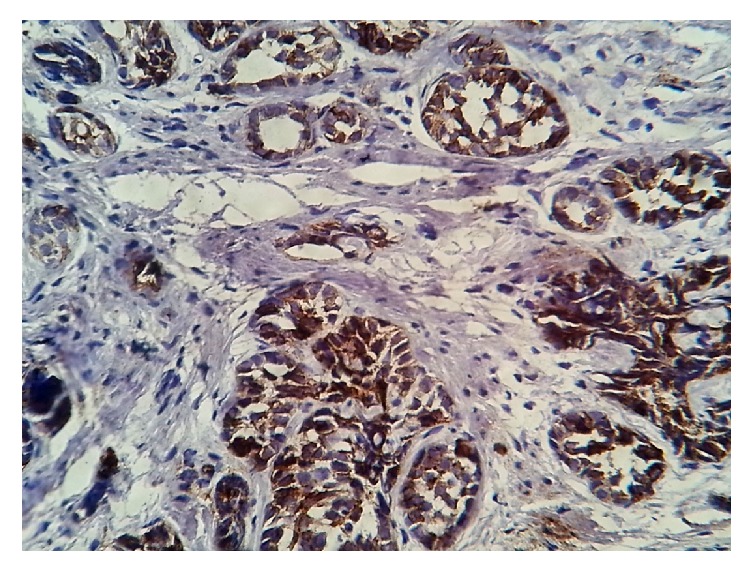
Immunostaining with CD 117: the adenoid cystic carcinoma cells are positive.

**Figure 4:**
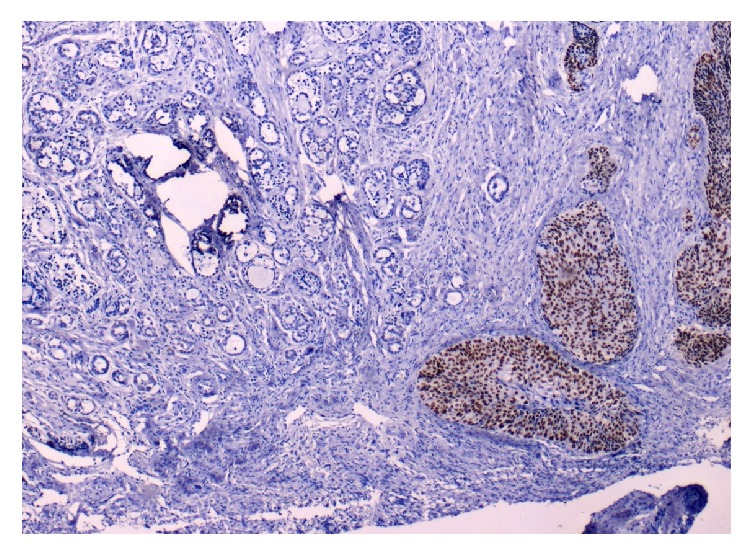
Immunostaining with P63: squamous cell component is positive (right), while adenoid cystic component is negative (left).

A deep cervical biopsy was performed. Histopathological examination of the sampled material showed multiple fragments, some of which were composed of a squamous mucosa with a squamous carcinoma in situ (Figure 1). Other fragments contained proliferation made of islands and nests punctuated by round spaces, showing a cribriform pattern, and small ductal structures formed by one or two layers of tumor cells. Some spaces contained eosinophilic secretion. Tumor cells were small, basaloid with hyperchromatic nuclei and scant cytoplasm (Figure 2). Focal palisading of the tumor cells was observed at the periphery of the islands. On immunohistochemical study, ACC tumor cells showed positive staining with CD117 (Figure 3). P63 was negative in the adenoid cystic component and diffusely positive in the squamous cell carcinoma in situ (Figure 4). A diagnostic of adenoid cystic carcinoma associated with squamous cell carcinoma in situ of the uterine cervix was made.

**Case 2 .**

A 60-year-old woman had intermittent purulent watery discharge and atypical genital bleeding of one year duration. She had no significant prior medical history. The speculum examination showed a cauliflower-like ulcerated tumor, arising from the portio, involving all fornices, and extending to the upper third of the vagina. A cervical biopsy was performed. Histological study of the sampled material showed proliferation of basaloid cells that had a cribriform and tubular pattern, and contained eosinophilic secretions that were positive with PAS and Alcian blue. Other areas showed an invasive squamous cell carcinoma. On immunohistochemical study, the ACC component was diffusely positive for CD117 and weakly and focally positive for P63; the SCC component was negative for CD117 and diffusely positive for P63. The patient was diagnosed as having adenoid cystic carcinoma associated with SCC infiltrating the uterine cervix. Chest X-ray was normal. Computed tomography of pelvis revealed a cervical mass measuring 6 × 5 × 5 cm with bilateral parametrial extension. The patient was staged IIb according to classification of the International Federation of Gynecology and Obstetrics (FIGO). She was treated with 46 Gy of photon external-beam radiotherapy and concurrent chemotherapy. Subsequent clinical examination revealed a residual cervical tumor measuring 3,5 cm. Total hysterectomy with bilateral adnexectomy was performed. Pathologic examination of the surgical specimen found scattered residual foci of SCC associated with a 2 × 2 cm adenoid cystic carcinoma, with negative surgical margins. The postoperative period was uneventful. The patient has been well, without evidence of recurrent disease for 2 years after the initial treatment.

## 3. Discussion

We present two new cases of coexisting adenoid cystic carcinoma with HSIL and invasive squamous cell carcinoma (SCC), respectively. This is a rare occurrence, in a recent study, Shi et al. [12] reviewed the literature and found only 27 cases of cervical ACC associated with SSC, including the three cases reported in their study. Nevertheless, the existence of such associations may suggest a common cellular origin and/or causative agent. In fact, some immunohistochemical evidence suggests that ACC develops from the multipotent reserve cell layer of the cervical epithelium that can show squamous or glandular differentiation [11]. Also, Shi et al. [12] demonstrated an integration of high-risk Human Papilloma Virus (HPV) in ACC and SCC, indicating that HPV may play a significant role in tumor pathogenesis.

ACC of the cervix generally occurs in older women in their sixth and seventh decades [6]. Clinically, it presents as a hard palpable mass that can be ulcerated or friable. The main symptom is usually vaginal bleeding [13]. These clinical features are consistent with those of the cases presented here, our patients were aged 74 and 60 years, and they both presented with vaginal bleeding and had a palpable mass.

Histologically, both our patient had characteristic features of ACC; these tumors are composed of fairly uniform, small basaloid cells with scanty cytoplasm and rounded or angulated hyperchromatic nuclei [6]. The cells are arranged in islands of crowded cells with little cytoplasm in a cribriform pattern with central hyaline or mucinous material. Other less frequent patterns may be observed, including trabecular, tubular, solid, or undifferentiated patterns, often with surrounding hyaline material. Lymphovascular invasion is frequent [10]. In our two patients, cribriform and tubular patterns were observed. However, neither case had necrosis or lymphovascular invasion.

The differential diagnosis includes adenoid basal carcinoma (ABC) and basaloid squamous cell carcinoma (SCC). ABC is believed to have a much less aggressive course than ACC [12]. The differential diagnosis is based on morphology; cellular pleomorphism, mitoses, necrosis, and stromal hyalinization are observed in ACC; in contrast, ABC lacks intraluminal hyaline material frequently present in ACC, and tumor cells have less pleomorphic nuclei and show less mitotic activity [14]. Besides, ABC can be distinguished by the absence of CD117 immunostaining [15]. Both our patients had cellular proliferation with moderate atypia, mitoses, and mucoid eosinophilic material in the lumen. They had diffuse immunostaining with CD 117. On the other hand, ACC (especially the solid variant) can be differentiated from basaloid SCC by immunohistochemistry, as squamous cell carcinoma stains strongly and positively with P63 and CK5/6 while ACC cells are generally negative [12, 16] this pattern was observed in the present cases.

ACC of the cervix seems to be more aggressive than squamous cell carcinoma with higher tendency to local and metastatic recurrence [17, 18]. The solid subtype has a worse prognosis in terms of the development of distant metastases and overall survival. The cribriform subtype has shown greater local aggressiveness and a poorer survival rate as compared with the tubular subtype [8]. Other specific tumor features that are connected to high risk of recurrence and mortality in stage Ib cervical cancers in general should be considered in ACC. They include large tumor diameter, deep stromal invasion, and the presence of tumor in the capillary lymphatic spaces found on microscopic examination [18]. The outcome in advanced disease (stage III and IV) had been reported to be invariably poor [19]. Clinical follow-up was available in the second patient, who was classified stage IIb of the disease, but had a 2-year clinical and imaging follow-up without disease recurrence.

The treatment of cervical ACCs comprises surgery and radiation therapy and/or chemotherapy which are usually recommended as an adjuvant treatment [17, 19]. ACC of cervix is considered as a radiosensitive tumor and better results have been reported in early stages patients that have been treated with adjuvant radiotherapy, as compared to those seen in cases where surgery has been done alone [20, 21]. Dixit et al. recommended surgery with adjuvant chemoradiotherapy for early stage and chemoradiotherapy in advanced stage [22].

## 4. Conclusion

ACC of the cervix is a rare, particularly aggressive neoplasm. It requires enhancement of postoperative treatment regimens and careful follow-up and thus needs to be distinguished from other tumors with similar histologic aspects.

The association of ACC with squamous cell carcinoma suggests a common origin, and further studies are required to explore the histogenesis of ACC in the uterine cervix.
